# P01.15. Multiple Myeloma, chemotherapy and curcumin

**DOI:** 10.1186/1472-6882-12-S1-P15

**Published:** 2012-06-12

**Authors:** E Panutich, H Zwickey

**Affiliations:** 1 National College of Natural Medicine, Portland, USA

## Purpose

Multiple Myeloma is one of the hematological malignancies that is caused by an unregulated proliferation of a single clone of plasma cells in the bone marrow leading to anemia, thrombocytopenia, bone destruction and pathological fractures. Accumulation of M-protein in the plasma suppresses normal level of immunoglobulins. Chemotherapy is based on the combination of alkylating agents, use of high dose of corticosteroids, combination of antimitotic agents, proteosome inhibitors and immunomodulators. Large numbers of people with Multiple Myeloma are not sensitive enough to standard chemotherapy or develop severe side effects that lead to termination of conventional treatment. Curcumin is a polyphenolic compound of turmeric that has diverse pharmacological effects including anti-inflammatory, antioxidative, antiproliferative and angiogenic activities. Several studies showed that curcumin can prevent drug resistance and increase the effect of several chemotherapeutic agents.

## Methods

We utilized a comprehensive review of mechanisms and biological activities of curcumin as a chemosensitizer and anticancerous compound through PubMed and other databases. Also, we analysed the reports and clinical trials which have examined specific effects of curcumin on different malignancies.

## Results

The research within the last several years *in vitro* and *in vivo* (animal models) showed that curcumin can sensitize cancer cells to different chemotherapeutic agents that are commonly used for Multiple Myeloma treatment. Curcumin up regulates caspase family proteins and down regulates antiapoptotic genes such as Bcl-2 and Bcl-X. The mechanisms by which curcumin may mediate chemotherapy include down regulation of various growth regulatory pathways and specific genetic targets such as NF-kB and COX2. Another property of curcumin is the ability to activate NRF2 and induce the expression of antioxidant enzymes that are shown to protect normal tissue and organs during chemotherapy.

## Conclusion

Preclinical studies on Curcumin and chemotherapeutic agents are expected to lead to clinical trials that will improve the treatment outcome for the patients with Multiple Myeloma.

